# Radiotherapy for Elderly Patients Aged ≥75 Years with Clinically Localized Prostate Cancer—Is There a Role of Brachytherapy?

**DOI:** 10.3390/jcm7110424

**Published:** 2018-11-08

**Authors:** Hideya Yamazaki, Koji Masui, Gen Suzuki, Satoaki Nakamura, Norihiro Aibe, Daisuke Shimizu, Tatsuyuki Nishikawa, Haruumi Okabe, Ken Yoshida, Tadayuki Kotsuma, Eiichi Tanaka, Keisuke Otani, Yasuo Yoshioka, Kazuhiko Ogawa

**Affiliations:** 1 Department of Radiology, Graduate School of Medical Science, Kyoto Prefectural University of Medicine, 465 Kajiicho Kawaramachi Hirokoji, Kamigyo-ku, Kyoto 602-8566, Japan; mc0515kj@koto.kpu-m.ac.jp (K.M.); gensuzu@koto.kpu-m.ac.jp (G.S.); satoaki@nakamura.pro (S.N.); a-ib-n24@koto.kpu-m.ac.jp (N.A.); dshimizu@koto.kpu-m.ac.jp (D.S.); 2 Department of Radiology, Ujitakeda Hospital, Uji-City, Kyoto 611-0021, Japan; tnishikawa201809@yahoo.co.jp (T.N.); h-okabe@takedahp.or.jp (H.O.); 3 Department of Radiation Oncology, National Hospital Organization Osaka National Hospital, 2-1-14, Hoenzaka, Chuo-ku, Osaka 540-0006, Japan; rad113@osaka-med.ac.jp (K.Y.); tkotsuma-osaka@umin.net (T.K.); tanaka@onh.go.jp (E.T.); 4 Department of Radiation Oncology, Osaka University Graduate School of Medicine, Suita, Osaka 565-0871, Japan; ohtanik@radonc.med.osaka-u.ac.jp (K.O.); yasuo.yoshioka@jfcr.or.jp (Y.Y.); kogawa@radonc.med.osaka-u.ac.jp (K.O.)

**Keywords:** prostate cancer, high dose rate, low dose rate, brachytherapy, IG-IMRT, elderly

## Abstract

We compared radiotherapy outcomes between 241 elderly patients aged ≥75 years and 867 younger controls (age <75 years) with clinically localized prostate cancer. The elderly group showed an equivalent actuarial seven-year biochemical failure-free survival rate (7y-bNED) (94.9%) to the younger control group (96.4%, *p* = 0.593). The incidence of late genitourinary (GU) and gastrointestinal (GI) toxicities grade ≥2 was also similar between the elderly and younger cohorts, while no grade ≥4 adverse events occurred. We also examined the role of brachytherapy (BT) in the elderly group, in comparison with image-guided intensity-modulated radiotherapy (IG-IMRT). BT showed superior 7y-bNED (94.1%) than IG-IMRT (84.6%, *p* = 0.0183) in elderly patients, which was 100% (100% for BT and 100% for IG-IMRT, *p* > 0.999) for the low-risk group, 94.6% (92.8% and 100%, *p* = 0.203) for the intermediate-risk group, and 80.5% (91.2% and 73.6%, *p* = 0.0195) for the high-risk group. BT showed higher GU toxicity and equivalent GI toxicity to IG-IMRT. In conclusion, elderly patients showed bNED and toxicity that were equivalent to those observed in younger controls, and BT is a plausible option also for healthy elderly with potential to improve bNED, with higher but acceptable GU toxicity.

## 1. Introduction

Prostate cancer is the most frequently diagnosed cancer in men, with the exception of skin cancer in developed countries [1], and the frequency of prostate cancer may increase with longer life expectancies [2]. Although, the current common curative treatment options include radical prostatectomy, external beam radiotherapy (EBRT), and interstitial brachytherapy (BT), the standard treatment for elderly patients is vague [3,4]. One reason is due to the heterogeneous nature of the elderly population, and it is difficult to select the best treatment for elderly patients, who have a high prevalence of comorbidities. Since the growth of prostate cancer is generally slow, and all interventions adversely affect quality of life to some extent, conservative management may be the best option for fragile elderly patients with prostate cancer [3,4]. In agreement, the incidence of prostate cancer death was not high in patients with prostate cancer who were treated conservatively [5,6,7].

In contrast, we often encounter healthy elderly patients with local prostate cancer who can endure aggressive standard treatment. Previous reports support aggressive treatments such as hormonal therapy, surgery, external radiation, and BT alone or in combination for healthy elderly men, especially those with high-risk prostate cancer [4,8,9,10,11,12]. Thus, after a careful evaluation of the nature of the cancer and comorbidities, there is room for aggressive, curative treatments that can maintain a patient’s quality of life.

BT has a higher conformality (better dose distribution with sharp dose-off gradient) than that of EBRT, potentially improving the therapeutic ratio by allowing higher doses to the tumor cells without elevating dose to surrounding normal tissue. The improved biochemical control shown in trials resulted in the inclusion of BT alone or EBRT plus BT as a standard treatment option supported by the National Comprehensive Cancer Network (NCCN) and American Society of Clinical Oncology (ASCO) guidelines for intermediate- to high-risk prostate cancer [3,13,14]. However, little evidence was accumulated about BT for elderly patients due to the fearful and fragile nature of the elderly. Since we first implemented BT over 20 years ago, we explored the role of BT and modern EBRT (image-guided intensity-modulated radiotherapy; IG-IMRT) not only for younger patients, but also for healthy elder patients [15,16,17,18,19,20,21]. Then, we thought that BT could be a good option for healthy elderly individuals and made an analysis on a combined database [20,21]. This study aims to compare the results of elderly patients to those of younger counterparts in a database combined for this assessment [20,21], and to clarify the potential of BT for elderly men diagnosed with localized prostate cancer.

## 2. Methods

### 2.1. Patients

Between 1994 and 2013, 1108 patients were treated with radiotherapy for curative intent and were the subjects of this study. We included patients with clinical classification of malignant tumors (tumor/node/metastasis (TNM) stage T1–4 and N0M0, with histology-proven adenocarcinoma, who were treated with high-dose-rate BT (HDR-BT) monotherapy (*n* = 352; 172 from Osaka University and 180 from Osaka National Hospital) or low-dose-rate BT (LDR-BT) with or without EBRT (*n* = 486, from Kyoto Prefectural University of Medicine), or IG-IMRT (*n* = 270, from Ujitakeda Hospital). Criteria also included availability and accessibility of pretreatment prostate-specific antigen (initial PSA = iPSA) level, Gleason score sum (GS), and T classification data on, and a minimum of one-year follow-up for surviving patients or until death. Of the 1128 included patients, 20 were excluded due to loss to follow-up at less than one year or because of missing data.

In total, 241 elderly patients were compared to 867 younger counterparts, and the patients’ clinical characteristics are shown in Table 1. Patients were staged according to the National Comprehensive Cancer Network (NCCN) 2015 risk classification as follows: low, T1–T2a and GS 2–6 and iPSA <10 ng/mL; intermediate, T2b–T2c or GS 7 or PSA 10–20 ng/ml; high, T3a–T4 or GS 8–10 or PSA >20 ng/mL [3]. PSA failure was defined using the Phoenix definition (nadir, +2 ng/mL) or as the start of salvage hormonal therapy. The Common Terminology Criteria for Adverse Events version 4.0 Toxicity was applied for toxicity analyses. All patients provided informed written consent. This study was conducted in accordance with the Declaration of Helsinki and institutional review board (IRB) permission at each institution.

### 2.2. Treatment Planning

#### 2.2.1. Brachytherapy (BT)

The BT regimens contained low-dose-rate interstitial BT (LDR-BT) with or without external beam radiotherapy (EBRT) and high-dose-rate interstitial BT (HDR-BT) monotherapy.

##### Low-Dose-Rate Interstitial BT (LDR-BT) with or without External Beam Radiotherapy (EBRT)

The intraoperative permanent I-125 implantation technique (The OncoSeed model 6711; General Electric Healthcare, Barrington, IL, USA) was previously described in detail [15,16]. We used Inter-Plan version 3.4 (ELEKTA, Stockholm, Sweden) for treatment planning, and the prescription dose to the clinical target volume (prostate) was 145 Gy (LDR-BT alone, *n* = 418, *n* = 60 in elderly) or 110 Gy (LDR-BT with EBRT, *n* = 68, *n* = 10 in elderly). We added EBRT to each patient with T3a or a Gleason score sums greater than or equal to eight or a Gleason score sum of seven (4 + 3), but not for a Gleason score sum of seven (3 + 4) cases, in addition to LDR-BT.

##### High-Dose-Rate Interstitial Brachytherapy (HDR-BT) Monotherapy

The details of the HDR-BT monotherapy technique and its use in previous studies were described elsewhere [17,18]. The major prescribed dose was 45.5 Gy per seven fractions (*n* = 86, *n* = 19 in elderly), 54 Gy per nine fractions (*n* = 111, *n* = 24 in elderly), and 49 Gy per seven fractions (*n* = 148, *n* = 38 in elderly), or other (36–38 Gy per four fractions, *n* = 7, *n* = 4 in elderly). We aimed for a tumor biologically effective dose (BED) of 240–270 Gy (α/β ratio = 1.5 Gy) and for a normal tissue BED of 144–162 Gy (α/β ratio = 3.0 Gy), with a difference that was expected to constitute an enhanced therapeutic ratio. The treatment machine was the microSelectron-HDR^®^ (Nucletron an Elekta Company, Veenendaal, The Netherlands, Elekta AB, Stockholm, Sweden).

#### 2.2.2. Image-Guided Intensity-Modulated Radiotherapy (IG-IMRT)

The IG-IMRT technique with helical tomotherapy was described elsewhere [19]. Briefly, we used computed tomography (CT; slice thickness of 2 mm in a supine position) and magnetic resonance imaging (MRI) data for treatment planning. Fusion of MRI images (T1w and T2w) were used for meticulous radiotherapy planning. The clinical target volume (CTV) was defined for the prostate and proximal seminal vesicles or prostate only in the low-risk group (Damico’s classification: stage, T1c; Gleason score <7; and PSA <10 ng/mL). We used a D95 (95% of planning target volume (PTV) received at least the prescribed dose) of 74.8 Gy/34 fractions (2.2 Gy/fraction, *n* = 104) for intermediate- and high-risk patients and 72.6 Gy/33 fractions (*n* = 23) for low-risk patients who were treated between June 2007 and June 2009. We modified the prescribed dose, reduced to 74 Gy/37 fractions (2 Gy/fraction, *n* = 118), for the high- and intermediate-risk groups and to 72 Gy in 36 fractions (*n* = 25) for the low-risk group from June 2009 to September 2013.

### 2.3. Statistical Analysis

The StatView 5.0 statistical software package (SAS Institute, Cary, NC, USA) was used for statistical analyses. Percentages were analyzed using the chi-square test, and Student’s *t*-test was used for normally distributed data. The Mann–Whitney U-test for skewed data was used to compare means or medians. The Kaplan–Meier method was used to analyze biochemical control rate, survival, and accumulated toxicity, and comparisons were made using the log-rank test. Cox’s proportional hazard model was used for uni- and multivariate analyses. A *p*-value <0.05 was considered as statistically significant.

## 3. Results

### 3.1. Biochemical Control, Survival, and Toxicity between Elderly Patients and Younger Controls

The median follow-up duration for the entire cohort was 87 (range 12–216) months, with a minimum of one year for surviving patients or until death. A comparison of patient backgrounds is shown in Table 1. The elderly group showed advanced disease (higher T category, higher iPSA, higher Gleason score sum, higher ratio of intermediate–high-risk groups in NCCN), with a higher incidence of hormonal therapies than younger controls.

**Figure 1:**
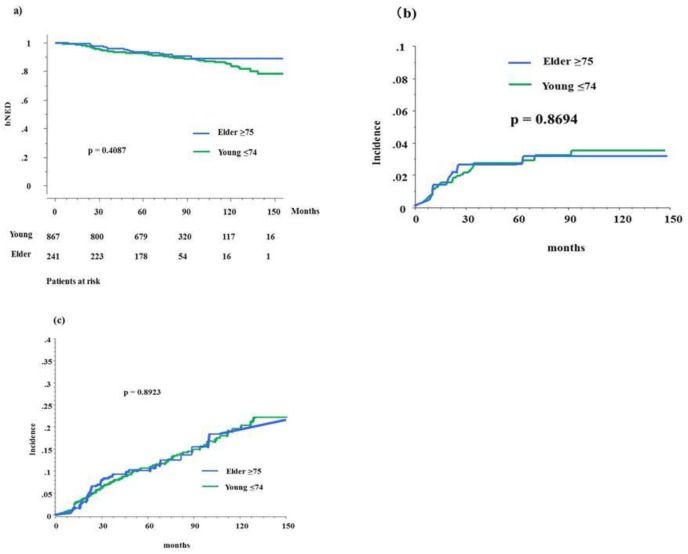
Biochemical control rates and accumulated incidence of toxicity between elder and younger counterparts. (**a**) Biochemical control rates between elderly (≥75) and control (young <75) in total population. (**b**) Accumulated incidence of gastrointestinal toxicity grade ≥2 between elderly and control in total population. (**c**) Accumulated incidence of genitourinary toxicity grade ≥2 between elderly and control in total population. bNED = no biochemical evidence of disease.

In the elderly group, 19 (7.9%) patients developed biochemical failure, compared with 96 (11.07%) patients in the control group. The actuarial seven-year biochemical failure-free survival rate (bNED) was 89.8% (95% confidential intervals = 95% CIs, 87.5–92.0%) and 90.6% (86.3–96.9%, *p* = 0.4087, Figure 1) (hazard risk 1.231, 95% CIs = 0.751–2.018, *p* = 0.4098) in the elderly and control groups, respectively. The bNED was 95.4% (100% for elderly and 94.7% for control, *p* = 0.4771) for the low-risk group, 91.2% (94.6% and 90.2%, *p* = 0.1692) for the intermediate-risk group, and 84.2% (80.5% and 84.8%, *p* = 0.8729) for the high-risk group. There was a significant difference in biochemical control rates among the three risk groups (*p* < 0.0001).

As shown in Table 2, our univariate analysis showed that the predictors of biochemical control included T classification (T1–2 vs. T3–4), Gleason score sum (≤7 vs. ≥8), a higher baseline PSA (<20 vs. ≥20), and treatment modality (IG-IMRT vs. BT). In our multivariate Cox regression analysis, higher iPSA and treatment modality (BT vs. IG-IMRT) remained significant factors for improving biochemical control.

In the elderly group, no patients developed distant metastasis, compared with 18 (17 bone and one lung; 2.1%) patients in the control group. The seven-year distant metastasis-free survival values were 100% (95% CIs = 100–100%) and 98.6% (97.8–99.4%, *p* = 0.052) in the elderly and control groups, respectively.

The overall seven-year survival rate was 87.6% (95% CIs = 82.4–92.9%) and 96.9% (95.4–98.3%, *p* < 0.0001) in the elderly and control groups (hazard risk (HR) = 0.339, 95% CIs = 0.201–0.571, *p* < 0.0001), respectively, and 97.3% (96.6% and 97.5%, *p* = 0.3859) for the low-risk group, 96.2% (90.5% and 98.0%, *p* = 0.0164) for the intermediate-risk group, and 91.4% (79.6% for elderly and 94.9% for control groups, *p* = 0.0010) for the high-risk group. There were statistically significant differences in overall survival rates among the three risk groups (*p* = 0.0172).

As there were only three prostate cancer-related deaths in this cohort (three high-risk patients), the seven-year cause-specific survival rate was 99.7% (100% in elderly and 99.7% in control groups, *p* = 0.175).

Table 3 shows the incidence of maximal late gastrointestinal (GI) and genitourinary (GU) toxicities between elderly and younger cohorts. No grade ≥4 late complications were observed in either group. Grades 1, 2, and 3 late GI toxicities occurred in 18 (7%), seven (3%), and zero patients in the elderly group and in 80 (9%), 22 (3%), and six (0.7%) participants in the control group, respectively (*p* = 0.4722). Late GU toxicities of grades 1, 2, or 3 occurred in 71 (29%), 29 (12%), and two (1%) patients in the elderly group and in 260 (30%), 114 (13%), and 13 (1.5%) patients in the control group (*p* = 0.7958), respectively. The incidence of grade ≥2 GU toxicity was 13.6% at seven years in the elderly group and 14% in the control group (Figure 1c, *p* = 0.9891), and that of GI toxicity at seven years was 3.1% and 3.3% in the elderly and control groups, respectively (Figure 1b, *p* = 0.8694).

### 3.2. Biochemical Control and Survival in Elderly Patients after BT or IG-IMRT

A comparison of the patient backgrounds is shown in Table 4. The IG-IMRT group was associated with a more advanced disease (higher T category, higher iPSA, higher Gleason score sum, and higher ratio of high-risk groups in NCCN) and a greater prevalence of hormonal therapy than the BT group. Table 5 shows the comparison between LDR-BT and HDR-BT. HDR-BT was used to treat advanced disease more often than LDR-BT.

**Figure 2:**
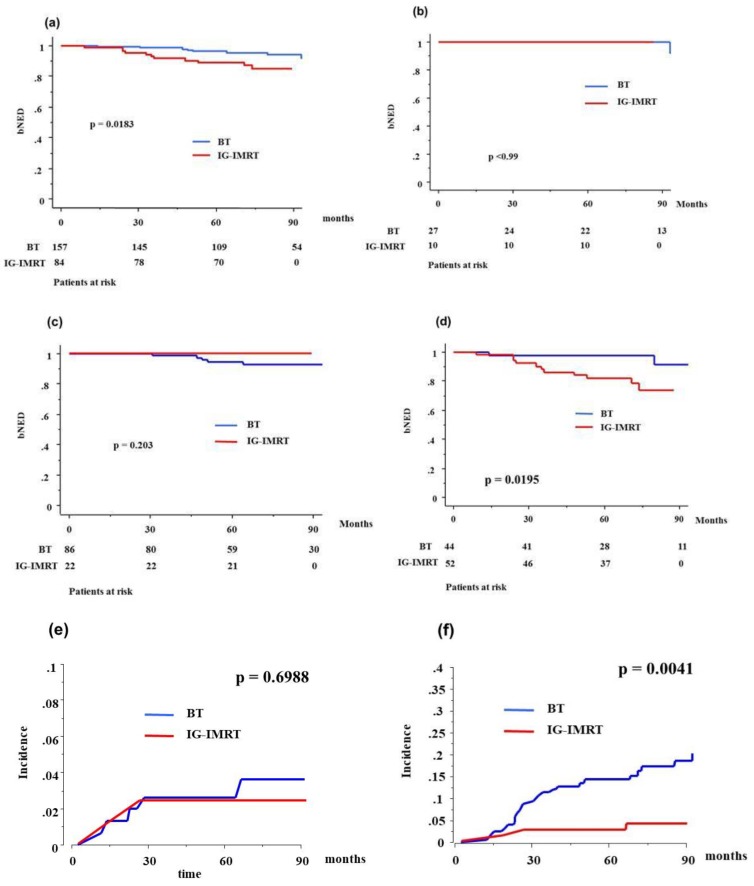
Biochemical control rates and accumulated incidence of grade ≥2 toxicity between brachytherapy (BT) and image-guided intensity-modulated radiotherapy (IG-IMRT) in elderly patients: (**a**) biochemical control rates between BT and IG-IMRT in elderly; (**b**) low-risk group; (**c**) intermediate-risk group; (**d**) high-risk group; (**e**) accumulated incidence of gastrointestinal toxicity grade ≥2 between BT and IG-IMRT; (**f**) accumulated incidence of genitourinary toxicity grade ≥2 between BT and IG-IMRT. bNED = no biochemical evidence of disease.

**Figure 3:**
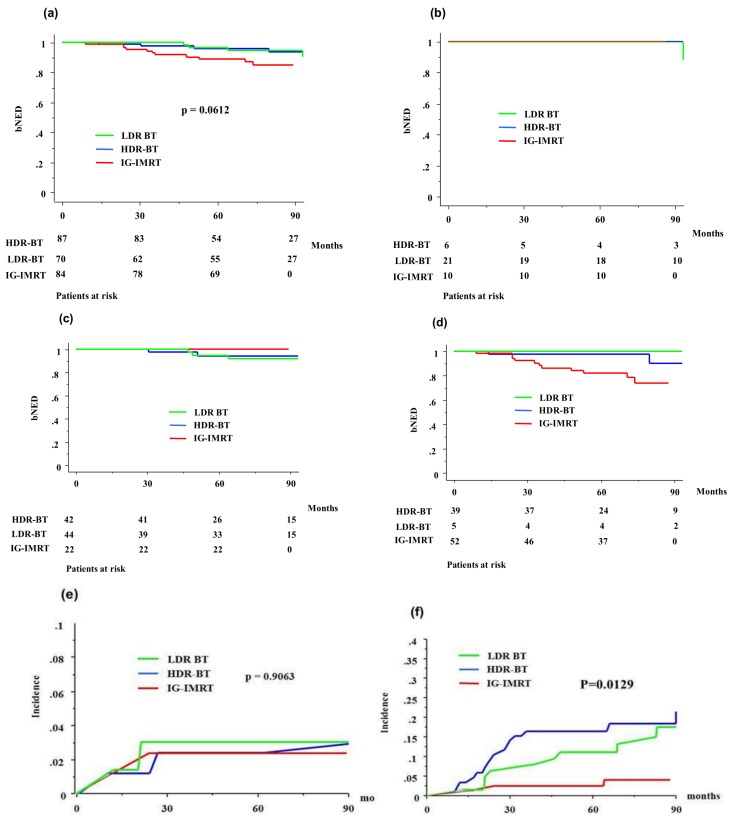
Biochemical control rates and accumulated incidence of grade ≥2 toxicity among IG-IMRT, high-dose-rate BT (HDR-BT), and low-dose-rate BT (LDR-BT) in elderly patients: (**a**) total population; (**b**) low-risk group; (**c**) intermediate-risk group; (**d**) high-risk group; (**e**) accumulated incidence of gastrointestinal toxicity grade ≥2; (**f**) accumulated incidence of genitourinary toxicity grade ≥2. bNED = no biochemical evidence of disease.

In the elderly group, eight (5.09%) who were treated with BT (four in HDR-BT and four in LDR-BT) developed biochemical failure, compared with 11 (13.09%) patients in the IG-IMRT group. The actuarial seven-year bNED was 94.1% (95% CIs = 89.7–98.5%) and 84.6% (75.8–93.4%, *p* = 0.0183, Figure 2) (hazard risk 0.336, 95% CIs = 0.130–0.870, *p* = 0.0246) in the BT (93.3% in HDR-BT and 94.9% in LDR-BT, *p* = 0.8985, Figure 3) and IG-IMRT groups, respectively, and 100% (100% for BT (100% in HDR-BT and 100% in LDR-BT, *p* = 0.8985) and 100% for IG-IMRT, *p* > 0.999) for the low-risk group, 94.6% (92.8% (94.4% in HDR-BT and 91.8% in LDR-BT, *p* = 0.7817) and 100%, *p* = 0.203) for the intermediate-risk group, and 80.5% (91.2% (89.9% in HDR-BT and 100% in LDR-BT, *p* = 0.613) and 73.6%, *p* = 0.0195) for the high-risk group. There was a significant difference in the biochemical control rate among the three risk groups (*p* = 0.010).

The overall seven-year survival rate was 89.2% (95% CIs = 83.5–94.9%) and 81.4% (66.9–95.9%, *p* = 0.6634) (HR = 1.544, 95% CIs = 0.561–4.246, *p* = 0.4003) in the BT (83.3% in HDR-BT and 95.3%, *p* = 0.0380) and IG-IMRT cohorts, respectively, and 96.6% (95.2% (75% in HDR-BT and 100% in LDR-BT, *p* = 0.021) and 100%, no *p*-value available) for the low-risk group, 90.5% (90.3% (94.4% in HDR-BT and 91.8% in LDR-BT, *p* = 0.7817) and 90.9%, *p* = 0.7253) for the intermediate-risk group, and 79.6% (82.7% for BT (82.9% in HDR-BT and 80% in LDR-BT, *p* = 0.6528) and 72.5% for IG-IMRT, *p* = 0.9627) for the high-risk group. There were statistically significant differences in overall survival rates among the three risk groups (*p* = 0.0314).

Table 6 and Table 7 show the incidences of maximal late GI and GU toxicities. No grade ≥4 late complications were observed in either group. The BT group showed a higher incidence of GU toxicity (*p* < 0.0001) and an equivalent incidence of GI toxicity (*p* = 0.9257), compared to the IG-IMRT group. HDR-BT showed an equivalent toxicity profile to LDR-BT (Table 5b). The incidence of grade ≥2 GU toxicity was 18.5% at seven years in the BT group and 3.9% in the IG-IMRT group (*p* = 0.0041), and that of GI toxicity was 2.4% and 3.6% in the BT and IG-IMRT groups, respectively (*p* = 0.6988, Figure 2). HDR-BT showed an equivalent late toxicity to LDR-BT both in GU and GI (Table 5, Figure 3).

## 4. Discussion

Due to the increased mortality rate in elderly patients, most elderly men with prostate cancer are expected to die from causes other than prostate cancer [5,6]. Therefore, observation is a sensible choice that avoids unnecessary treatment (overtreatment), elevated medical costs, and adverse events. Several randomized controlled trials and population-based studies revealed no survival benefit for active intervention with surgery and radiotherapy in patients with low-risk prostate cancer, compared with conservative management [5,6,7]. The guidelines recommend conservative management for patients with low-risk prostate cancer with life expectancies of less than 10 years [3,4]. However, clinicians tend to avoid conservative management, and only 20–35% of elderly men with low-risk prostate cancer choose observation as the initial therapy [11,22,23].

On the other hand, there is a concern about healthy elderly patients with high-risk prostate cancer, who are often undertreated. International recommendations state that elderly patients should be managed according to their individual health status and not according to age, and fit elderly patients should receive the same treatment as younger patients [4]. However, Lunardi et al. reported a 16% undertreatment rate in older patients >75 years of age, without any significant comorbidity [24]. These discrepancies may be due to the lack of high-quality evidence from studies such as randomized controlled trials [22,24] and specific survival data comparing treated and untreated elderly patients. Therefore, our data provide useful information to choose radiotherapy, especially brachytherapy, for both physicians and patients, and will help guide decisions regarding whether BT or other treatments, such as observation, should be selected.

This is the first study to examine the role of BT in elderly patients. Interstitial BT can be divided into permanent implantation, low-dose-rate (LDR) and temporary implantation, and high-dose-rate (HDR). Both therapies have established roles as curative treatments for localized prostate cancer as BT alone or EBRT plus BT [3,14], and EBRT plus BT is a major procedure for the intermediate–high-risk group. On the other hand, several authors used HDR-BT as a monotherapy and reported excellent outcomes for all risk groups [17,18,25,26,27]. If this could be confirmed, it would be the most efficient method of achieving good dose distribution with a high degree of conformity—even for adjacent tissue invasion (seminal vesicle or extracapsular extension)—and a short overall treatment time. However, the use of multiple fractions of HDR-BT is not a recommended procedure as a standard therapy to fragile elder patients because of its invasive nature and long treatment duration at present. It is also true that the search for the optimal HDR-BT schedule for prostate cancer remains a challenge, although single-dose HDR-BT has an attractive possibility [27].

Our data provided additional non-inferior biochemical data from elderly patients and younger counterparts and revealed the superior outcomes of BT compared to IG-IMRT in elderly patients, especially those of the high-risk group. The strengths of our study are the inclusion of a large population with more than 1100 patients and a long follow-up period, over which the clinical characteristics of BT and IG-IMRT were analyzed. In addition, the toxicity analysis showed that age did not increase acute or late urinary or bowel toxicities, which concur with the results of a previous study [28].

Our study has several limitations. Firstly, as it is a retrospective investigation of results from a few institutions, a longer follow-up of a larger number of patients or a randomized controlled trial is needed to achieve a definitive conclusion. Furthermore, our study lacks a comorbidity analysis. Although our patients retained good performance statuses of 0–1, comorbidities are common in elderly patients. Several studies indicated that comorbidity is an important factor for choosing the curative treatment modality in elder populations [16,19]. Thirdly, anesthesia is part of invasive procedures, including brachytherapy. Fortunately, we did not experience any moderate to severe toxicity. Only mild transient (a few hours) headaches were found in several patients. However, it is also a barrier to undergoing BT for elder patients. Lastly, our superior biochemical control did not translate into better survival data. Notably, no survival impact was observed among several prostate cancer studies, even with an improved biochemical control and aggressive treatment [5,6,7,29].

In conclusion, elderly patients showed equivalent biochemical control and toxicity profiles to younger counterparts, and BT showed a superior biochemical control rate than IG-IMRT in the elderly population, especially in the high-risk group, with elevated but acceptable GU toxicity.

## Figures and Tables

**Table 1:** Characteristics and treatment factors of patients.

Variables	Strata	Elder (Age ≥75) *n* = 241		Control (Age ≤74) *n* = 867		*p*-Value
		*N* or Median (Range)	(%)	*N* or Median (Range)	(%)	
Age		77 (75–86)		68 (45–74)		**NA**
T category	1	73	(30%)	350	(40%)	**0.008**
	2	124	(51%)	381	(44%)	
	3	44	(18%)	126	(15%)	
	4	0	(0%)	10	(1%)	
Pretreatment PSA	ng/mL	9.70 (1.971–245)		8.26 (1.4–658)		**0.0312**
Gleason score	≤6	82	(34%)	399	(46%)	**0.0005**
	7	93	(39%)	314	(36%)	
	≥8	66	(27%)	154	(18%)	
NCCN risk classification	Low	37	(15%)	232	(27%)	**0.0008**
	Intermediate	108	(45%)	341	(39%)	
	High	96	(40%)	274	(32%)	
Treatment modality	IG-IMRT	84	(35%)	186	(21%)	**<0.0001**
	Brachytherapy	157	(65%)	681	(79%)	
	HDR-BT	87	(36%)	265	(31%)	
	LDR-BT	70	(29%)	416	(48%)	
Hormonal therapy	Yes	159	(66%)	447	(52%)	**<0.0001**
	No	82	(34%)	420	(48%)	
Follow-up	Months	74 (14.5–161)		83.4 (12–216)		**<0.0001**

Bold values indicate statistical significance. PSA = prostate-specific antigen; NCCN = National Comprehensive Cancer Network; NA = not available; HDR-BT = high-dose-rate brachytherapy, LDR-BT = low-dose-rate brachytherapy; IG-IMRT = image-guided intensity-modulated radiotherapy.

**Table 2:** Univariate and multivariate analysis for biochemical control rate using Cox proportional hazards model.

Variable	Strata	Biochemical Control					
		Univariate Analysis			Multivariate Analysis		
		HR	95% CIs	*p*	HR	95% CIs	*p*
Age (years)	<75	1	(referent)	-	1	(referent)	-
	≥75	1.232	0.752–2.020	0.407	1.309	0.793–2.162	0.2923
T classification	T1–2	1	(referent)	-	1	(referent)	-
	T3–4	2.313	1.551–3.449	**<0.0001**	1.676	0.989–2.840	0.0548
Gleason score	<7	1	(referent)	-	1	(referent)	-
	≥8	0.605	0.398–0.919	**0.0186**	0.838	0.526–1.336	0.4584
Pretreatment PSA (ng/mL)	<20	1	(referent)	-	1	(referent)	-
	≥20	0.399	0.270–0.590	**<0.0001**	0.481	0.293–0.791	**0.0039**
NCCN risk classification	Low	1	(referent)	-	NA		
	Intermediate	1.642	0.907–2.973	0.1018			
	High	3.211	1.820–2.973	**<0.0001**			
Hormonal therapy	No	1	(referent)	-	1	(referent)	-
	Yes	0.832	0.574–1.205	0.3302	1.426	0.903–2.252	0.128
Treatment modalities	IG-IMRT	1	(referent)	-	1	(referent)	-
	BT	0.555	0.366–0.841	**0.0055**	0.603	0.391–0.929	**0.0219**

Bold values indicate statistical significance; Abbreviations: CIs = confidence intervals; HR = hazard ratio, NA = not available; PSA = prostate-specific antigen; NCCN = National Comprehensive Cancer Network.

**Table 3:** Late toxicities according to age.

Toxicities	Strata	Control (Age ≤74)		Elder (Age ≥75)		*p*-Value
		*n* = 867		*n* = 241		
Gastrointestinal	0	759	(88%)	216	(90%)	0.4722
	1	80	(9%)	18	(7%)	
	2	22	(3%)	7	(3%)	
	3	6	(0.7%)	0	(0%)	
Genitourinary	0	480	(55%)	139	(58%)	0.7958
	1	260	(30%)	71	(29%)	
	2	114	(13%)	29	(12%)	
	3	13	(1.5%)	2	(1%)	

**Table 4:** Characteristics and treatment factors of elderly patients according to modality.

Variables	Strata	IG-IMRT *n* = 84		Brachytherapy *n* = 157		*p*-Value
		*N* or Median (Range)	(%)	*N* or Median (Range)	(%)	
Age		77 (75–86)		76 (75–86)		**0.0127**
T category	1	23	(27%)	50	(32%)	0.0727
	2	39	(46%)	85	(54%)	
	3	22	(26%)	22	(14%)	
	4	0	(0%)	0	(0%)	
Pretreatment PSA	ng/mL	10.0 (4.3–245)		9.4 (1.9–98.6)		0.1644
Gleason score	≤6	23	(27%)	59	(38%)	**<0.0001**
	7	23	(27%)	70	(45%)	
	≥8	38	(45%)	28	(18%)	
NCCN risk classification	Low	10	(12%)	27	(17%)	**<0.0001**
	Intermediate	22	(26%)	86	(55%)	
	High	52	(62%)	44	(28%)	
Treatment modality	HDR-BT			87	(61%)	NA
	LDR-BT			70	(39%)	
Hormonal therapy	Yes	64	(76%)	95	(61%)	**0.0211**
	No	20	(24%)	62	(39%)	
Follow-up	Months	73.4 (24–90)		77.3 (14.5–161)		**0.0312**

Bold values indicate statistical significance. NA = not available; PSA = prostate-specific antigen; NCCN = National Comprehensive Cancer Network; HDR-BT = high-dose-rate brachytherapy; LDR-BT = low-dose-rate brachytherapy; IG-IMRT = image-guided intensity-modulated radiotherapy.

**Table 5:** Characteristics and treatment factors of elderly patients between HDR-BT and LDR-BT.

Variables	Strata	HDR-BT *n* = 87		LDR-BT *n* = 70		*p*-Value
		*N* or Median (Range)	(%)	*N* or Median (Range)	(%)	
Age		76 (75–86)		77 (75–83)		0.4342
T category	1	21	(24%)	29	(41%)	**<0.0001**
	2	45	(52%)	40	(57%)	
	3	21	(24%)	1	(1%)	
	4	0	(0%)	0	(0%)	
Pretreatment PSA	ng/mL	10.8 (1.97–98.6)		7.45 (3.56–26)		**0.0002**
Gleason score	≤6	26	(30%)	33	(47%)	**0.0024**
	7	38	(44%)	32	(46%)	
	≥8	23	(26%)	5	(7%)	
NCCN risk classification	Low	6	(45%)	21	(7%)	**<0.0001**
	Intermediate	42	(48%)	44	(63%)	
	High	39	(7%)	5	(30%)	
Hormonal therapy	Yes	72	(83%)	47	(67%)	**<0.0001**
	No	15	(17%)	23	(33%)	
Follow-up	Months	72.2 (20–161)		83.7 (14.5–143)		0.2622

Bold values indicate statistical significance. NA = not available; PSA = prostate-specific antigen; NCCN = National Comprehensive Cancer Network; HDR-BT = high-dose-rate brachytherapy, LDR-BT = low-dose-rate brachytherapy.

**Table 6:** Late toxicities according to modalities in elderly.

Toxicities	Strata	BT *n* = 157		IG-IMRT *n* = 84		*p*-Value
		*N* (%)		*N* (%)		
Gastrointestinal	0	140	(89%)	76	(90%)	0.9257
	1	12	(8%)	6	(7%)	
	2	5	(3%)	2	(2%)	
	3	0	(0%)	0	(0%)	
Genitourinary	0	70	(45%)	69	(82%)	**<0.0001**
	1	59	(38%)	12	(14%)	
	2	27	(17%)	2	(2%)	
	3	1	(1%)	1	(0.4%)	

Bold values indicate statistical significance. IG-IMRT = image guided intensity modulated radiotherapy; BT = brachytherapy.

**Table 7:** Late toxicities according to modalities in elderly.

Toxicities	Strata	HDR-BT *n* = 87		LDR-BT *n* = 70		*p*-Value
		*N* (%)		*N* (%)		
Gastrointestinal	0	76	(87%)	64	(91%)	0.6943
	1	8	(9%)	4	(6%)	
	2	3	(3%)	2	(3%)	
	3	0	(0%)	0	(0%)	
Genitourinary	0	42	(48%)	28	(40%)	0.3804
	1	28	(32%)	31	(44%)	
	2	16	(18%)	11	(16%)	
	3	1	(1%)	0	(0%)	

LDR-BT = low-dose-rate brachytherapy; HDR-BT = high-dose-rate brachytherapy.
